# Dental Patents

**Published:** 1854-10

**Authors:** 


### DENTAL PATENTS.

[From the Dental News Letter.]

Gentlemen:—Under the above caption an article appeared in the October number of the "News Letter," for a reply to which I have in vain looked through the last number; and, as the assumptions therein made seem to me incorrect and really inadmissible, please permit a few words in defence of a worthy class of men, than whom none are more entitled to our regard, respect, praise and gratitude.

Since Whitney, Fulton and Arkwright have lived, it seems almost superfluous to eulogize inventors as a class. In the words of another: "When their wonderful discoveries, and the great results to individuals and to nations which have followed from them, are contemplated, it is not difficult to realize the value of the splendid gifts which science, through their instrumentality, has bestowed upon man, nor to estimate the claims which the true inventor has upon society. He may truly be called the pioneer of civilization, the explorer of the unknown world of science and art." That any other opinion should prevail; that any attempt, however feeble, should be made to filch from him his well-earned meed of fame or profit, does indeed seem "passing strange."

But to our article. The writer's *radical* error consists in this assumption:

"The natural right that any man has in any discovery he may make in art or science, ONLY entitles him to *conceal* and use, as best he may, his invention for his own benefit."

The mistake is a grave one; for it affects the original, inalienable right of proprietorship, of which every individual is conscious. Does your natural right in personal property which you possess, allow you only to hide, or use in secret, that property, lest the first strong or cunning hand which coverts possession shall rob you of it? On the contrary, is it not your privilege to use that property at home and abroad, to use it openly and freely, and to defend yourself by force of arms, when necessary, from any attempt unlawfully to dispossess you? Indubitably, yes. To this latter position no sane man will offer other answer. And this property may be the horse which you have bought, the grain which you have grown, or the implement fashioned by your hand. No *lesser* right has the *inventor* to the product of his mental toil; no lesser wrong do you perpetrate upon him when you steal the subtle, intangible principle of his discovery, than when you rob him of the tangible levers, pulleys and wheels, which, combined, are the embodiment of that principle. This right is as sacred as any for which our fathers ever fought, or in defence of which you to-day would fight. Nor is this fact affected by the statement that "no decision has ever yet been made in the United States by which the right to a discovery or invention has been protected by damages against infringement by any other form of legal remedy than that provided by the *patent laws*,"—for upon this very principle of the inviolability of private property, is based the law which provides that property in patent rights shall pass, after a term of years, into possession of the public —as we shall see more fully hereafter. Upon this point I quote from Mr. Burke, late Commissioner of Patents, in his Report for 1845: "The fruits of his genius and his toil are constantly liable to be wrested from him by the unscrupulous and dishonest, who, too often countenanced by public opinion, are apt to regard the rights of the inventor as the fruits of a monopoly which it is a merit, instead of a wrong, to break down and destroy; and the more valuable the invention the more liable is the patentee to this species of invasion and injury, as there is more inducement held out to its perpetration. The stealthy thief and the midnight burglar are justly regarded as the pests and enemies of society, and are therefore seized and punished by penalties severe in proportion to the turpitude of their crimes; yet their depredations are committed on things which are made by law the subjects of property, and which may be acquired by industry or by purchase. The right of the inventor to his invention, in the judgement of all enlightened minds, cannot but be viewed as far more sacred than mere things of property. It is a mental creation, or rather the discovery of a principal or thing never before known to the world, and may be, as very many inventions have been, productive of countless blessings to the human family, affecting their destinies as individuals and as communities, through all time." As a MAN, then, holding property in the glorious realm of thought, the inventor stands before you to be judged fairly, as should be other men.

Again, the writer thus states his position:

"The law, looking to the justice due to the public as carefully as to the rights of particular individuals, leaves discoverers to their own unassisted resources for making for themselves an absolute and exclusive property of their inventions, and interpose, *only* for the benefit of *the public*, its assurance of a monopoly for such a period of time as it is supposed may operate as an inducement to reveal the secret at once for general use."

Not so. This is no contract in which the inventor lies, like a turtle in his shell, until coaxed by governmental bounty, to put out its head, soon to be cut off, while a kind public, in the background, await the result, to parcel out his body for their individual uses. More noble, far higher, is the position of government—for it assumes a voluntary *protectorate* of the *rights* of individuals in their property. It recognizes the absolute ownership of his invention by the inventor, and proposes to protect him in the exclusive right of that property, but says to him, "After you shall have exclusively used this property for the term of fourteen or twenty-one years, and thus been *remunerated* in advance, we, for the benefit of the body politic, and in exercise of the power given us to legislate for the *general* welfare, claim the same for public use." To this effect an able writer has said: "The necessities of the government are, in certain emergencies, supreme over the rights of its citizens, and, by virtue if its right of eminent domain, it can take the property of its citizens for the public use. But in all civilized countries, in which the immutable principles of morality and justice prevail, governments never take the property of their citizens and appropriate it to their own use without first giving an adequate compensation for it." Thus is clearly stated the position of the inventor. Though he does "at once reveal the secret," it is not for "general use" until the fourteen years have passed. He may refuse to sell rights; may choose to manufacture and dole out machines at the slowest rate—it is his *right* so to do, and government acknowledges it. When the writer assumed that "there is no legal recognition of any such absolute property in any invention," he supported directly, or by inference, an error. The principle is old as the hills and changeless as truth. As in this world of continents and islands, men claim their discoveries, although fellow-voyagers may next day come from the same or an opposite direction, and as truly discover the same land—so in the world of science and art, inventors are the bold navigators who steer out into unknown oceans, and ungenerous is that man who denies to them every right belonging to discoverers.

But hear our author again:

"The friends of dental patents have a marked affection for the shelter which they fancy is afforded them by the *higher* and *more honorable* warranty of copyright, than that which can be drawn from the more mechanical and less professional example of patent rights."

What arrant heresy is this! Are Fulton, Whitney, Watt and Arkwright protected by *patents* for their world-blessing inventions, less to be respected than Dickens, Bulwer, Prescott and Irving, vending their *copyrighted* books? Truly our author deserves a "patent" for *his* discovery that the true aristocracy of intellect consists in moulding tropes and metaphors with a goose-quill, while *plebian* minds may forge from steel and iron the snorting steeds with which we conquor time and space, or the jennies which spin clothing for a world. The *distinction* here is without any corresponding essential *difference* in principle. The right of the author to his book is at once acknowledged; but, owing to the flexibility of language allowing easy evasion, and other causes operating to the disadvantage of authors, their patents (called copyrights) are extended over more time before the public claims their work. As a general rule, the necessity of a book for public use, its immediate public utility and capacity for practical application, are not so readily discovered as are the advantages of some machine, the direct operation of which every man can *see*, and the *money-making* properties of which he comprehends at a glance. The book may be very good, but sell slowly—the machine, if good, soon comes into general use, for it is the shorter way of coining *dimes*. The readiness with which the facts and thories of a book may be borrowed or remodelled, materially affects the early necessity of taking it from the individual and passing it over to government. The gentleman thinks that his relations are very different toward the author who brings to him a copyrighted book, and the inventor who brings to him a patented invention ; but I do not see the difference. If " Professor Harris, Arthur or Austen" benefits me by authorship, it is after I have paid two, four or six dollars for his patented *book*—price being regulated by security of copyright, conscience of publisher, demand in the market, etc. If Mr. Dubs benefits me by invention, it is after I have paid three, four or five dollars for his patented *forcep*—price being regulated as above. Now, wherein consists the difference but in a *name?* In each case *mind* is the craftsman—the *commodities* offered for sale only differ. Whether the man spend years in preparing a useful *treatise*, or in perfecting a useful *machine*, he is alike entitled to our regard, alike proprietor of the result of such labor ; and in the latter mode *may* as greatly benefit his kind as in the former. The merit of the individual, then, consists in the value of his book, the utility of his invention, and is not settled in advance by any misconceived title of "honorable" or dishonorable applied to his department of mental labor.

Note by the Editor.—The Dr.'s views of Copy and Patentright coincide very much with our own. We know that it is not the popular side of the question—yet we have not by any argument yet presented been able to see any difference in the principle—both are monopolies—both are sustained by law, and unless a better cause can be shown than this, the Patentee should not be held as a quack or charlatan. These being our views, we place our ground of objection to patents on remedial agents, to that moral obligation we assume in taking upon us the practice of any branch of the healing art. That everything here should be free, because the preservation of life is a paramont obligation resting upon us all, hence all law recognizes the right to preserve our life—it is a sacred trust and cannot be lawfully taken from us unless for a greater public good—having forfeited our right by an infringement on that of others. The laws for our mutual benefit in associations may be of such a character that would forbid a patentee fellowship; for if he has united with his professional brethren for mutual improvement and the good of the profession, and from selfish considerations keeps his knowledge out of the general stock, it would appear just and proper for him to retire. This, however, should be provided for in the Constitution of the Society itself. If not, it might appear as an act of injustice to take such in and then turn them out at pleasure.

